# Uncommon yet critical: Pulmonary embolism in a 14-year-old Nigerian child: A case report

**DOI:** 10.1097/MD.0000000000039503

**Published:** 2024-09-13

**Authors:** Obuoma Umejuru Amaewhule, Ebbi Donald Robinson, Ugoeze Nneka Iloeje, Emmanuel Ovundah Nyeche, Victoria Ezinne Emeruwa, Faithful Miebaka Daniel

**Affiliations:** a Department of Pediatrics, Rivers State University, Port Harcourt, Nigeria; b Department of Radiology, Rivers State University Teaching Hospital, Port Harcourt, Nigeria; c Department of Internal Medicine/Cardiology, Federal Medical Center, Yenagoa, Nigeria; d Department of Internal Medicine, Rivers State University Teaching Hospital, Port Harcourt, Nigeria; e V. N. Karazin National University, Kharkiv, Ukraine; Community and Clinical Research Division, First On-Call Initiative, Portharcourt, Nigeria; f Community and Clinical Research Division, First On-Call Initiative, Kharkiv, Ukraine; Community and Clinical Research Division, First On-Call Initiative, Port Harcourt, Nigeria.

**Keywords:** angiography, case report, factor Xa inhibitor, pulmonary embolism, tissue plasminogen activator

## Abstract

**Rationale:**

Pulmonary embolism is a rare life-threatening condition in pediatric populations. Diagnosis is often challenging in resource-constrained settings suffering chronic shortages of specialist and diagnostic services. We report the prompt recognition and challenging management of pulmonary embolism in an adolescent presenting to a private specialist hospital in a resource-constrained country. Although, majority of the Nigerian population utilize private healthcare, most centers are not equipped with sophisticated radiological and advanced laboratory services. These services were outsourced to a recently equipped state-owned tertiary hospital.

**Patient’s concerns:**

We present the case of a 14-year-old female who presented to the hospital with complaints of sharp left-sided chest pain and palpitations of 1 week duration. She was well until a week prior to the presentation when she noticed a sharp pain in her chest on waking up that was severe enough to make her cry. She was also felt her heart racing fast. The chest pain seemed to have subsided until a day prior to hospital presentation when she had a repeat episode following dance practice, necessitating her coming to the hospital.

On examination at presentation, she was in painful distress, mildly pale, anicteric, acyanosed, with no peripheral edema. She had tachycardia, and her pulse was full volume, regularly irregular, and synchronous with peripheral pulses. Her blood pressure was 110/70 mmHg, and her apex beat was at the 5th left intercostal space, mid-clavicular line, non-heaving. Heart sounds 1 and 2 only were heard. The diagnosis was confirmed using a D-dimer assay, Echocardiography, and Computerized tomography pulmonary angiogram.

**Diagnosis:**

A diagnosis of pulmonary embolism was made.

**Interventions:**

The patient received pharmacological management using low molecular weight heparin, recombinant tissue plasminogen activator, and direct factor Xa inhibitor to manage and resolve the embolism.

**Outcomes:**

The embolus was resolved after months of anticoagulant therapy, as confirmed by serial echocardiography.

**Lessons:**

The case highlights the need for low-resource settings to address diagnostic limitations and emphasizes the importance of a multidisciplinary approach to managing pulmonary embolism cases. It also adds to the growing evidence of the effective role of pharmacological therapy in the management of pulmonary embolism.

## 1. Introduction

Pulmonary embolism is a condition that occurs when blood clot (thrombi) gets lodged in a major artery in the lungs.^[1]^ The clot is usually formed in the veins of the legs and travels up to the right side of the heart to partially or completely occlude a pulmonary artery.^[1]^ This prevents gaseous exchange and blood flow to the part of the alveoli distal to the obstruction. Pulmonary embolism is rare in children and even when present it is often a diagnostic dilemma. This is a report about a 14-year-old Nigerian girl who was diagnosed with pulmonary embolism, a life-threatening condition. It is a rare presentation in pediatric age groups, and this is the first case diagnosed for this age group in this private specialist center. Many advanced radiological and laboratory investigations were not available in this center and similar private hospitals that serve a majority of patients in the State. The patient was jointly managed by the only State-owned tertiary hospital recently equipped with the state-of-the-art diagnostics, and servicing over 5 million residents of the state. The case aims to raise awareness of this condition in pediatric age groups. It also highlights the need for stakeholders in low-resource settings to address diagnostic limitations and emphasize the importance of a multi-disciplinary approach to the management of such cases.

## 2. Case presentation

O.P. was a 14-year-old female who presented to the hospital with complaints of sharp left-sided chest pain and palpitations of 1 week duration. She was well until a week prior to the presentation when she noticed a sharp pain in her chest on waking up that was severe enough to make her cry. She was also able to feel her heart racing fast. The chest pain seemed to have subsided until a day prior to the hospital presentation when she had a repeat episode following dance practice, necessitating her coming to the hospital. There was no history of trauma, surgery or hospital stay requiring indwelling central venous catheters. She was not a known patient with sickle cell disease or thrombophilia, and was not previously diagnosed with congenital heart defects.

On examination at presentation, she was in painful distress, mildly pale, anicteric, acyanosed, with no peripheral edema. She had tachycardia, and her pulse was full volume, regularly irregular, and synchronous with peripheral pulses. Her blood pressure was 110/70 mmHg, and her apex beat was at the 5th left intercostal space, mid-clavicular line, non-heaving. First and second heart sounds only were heard.

**Figure 1.**
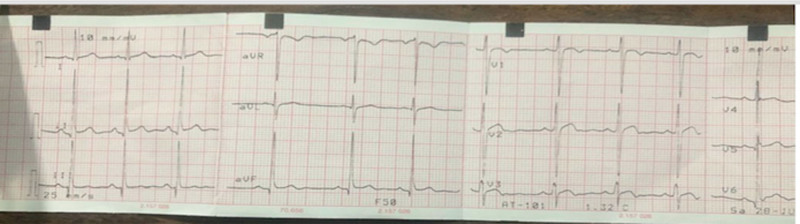
Electrocardiogram showing sinus arrhythmia and ST anomalies.

**Figure 2.**
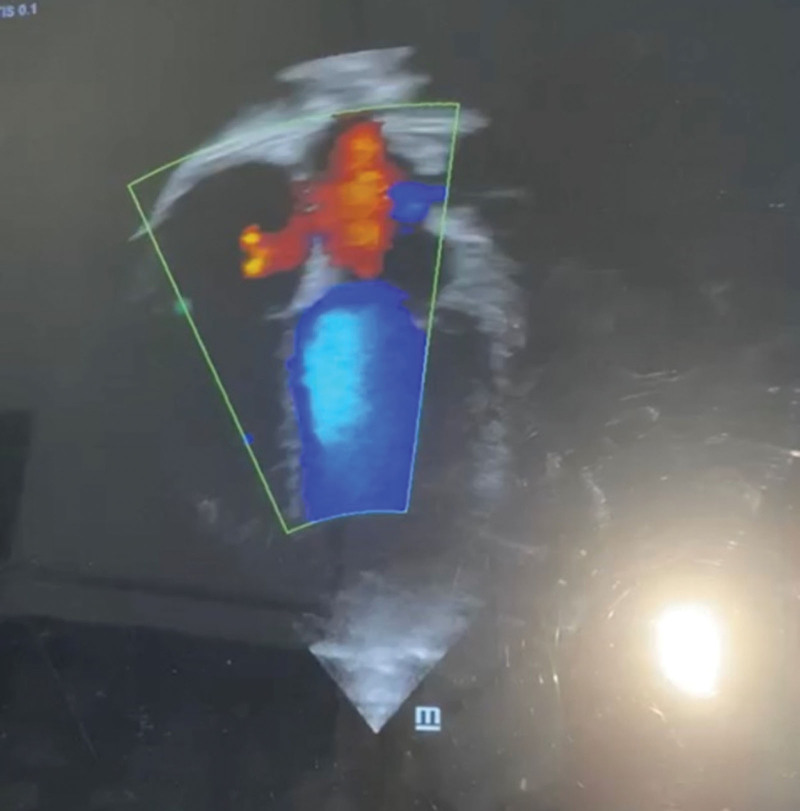
Echocardiographic image showing ostium secundum atrial septal defect.

**Figure 3.**
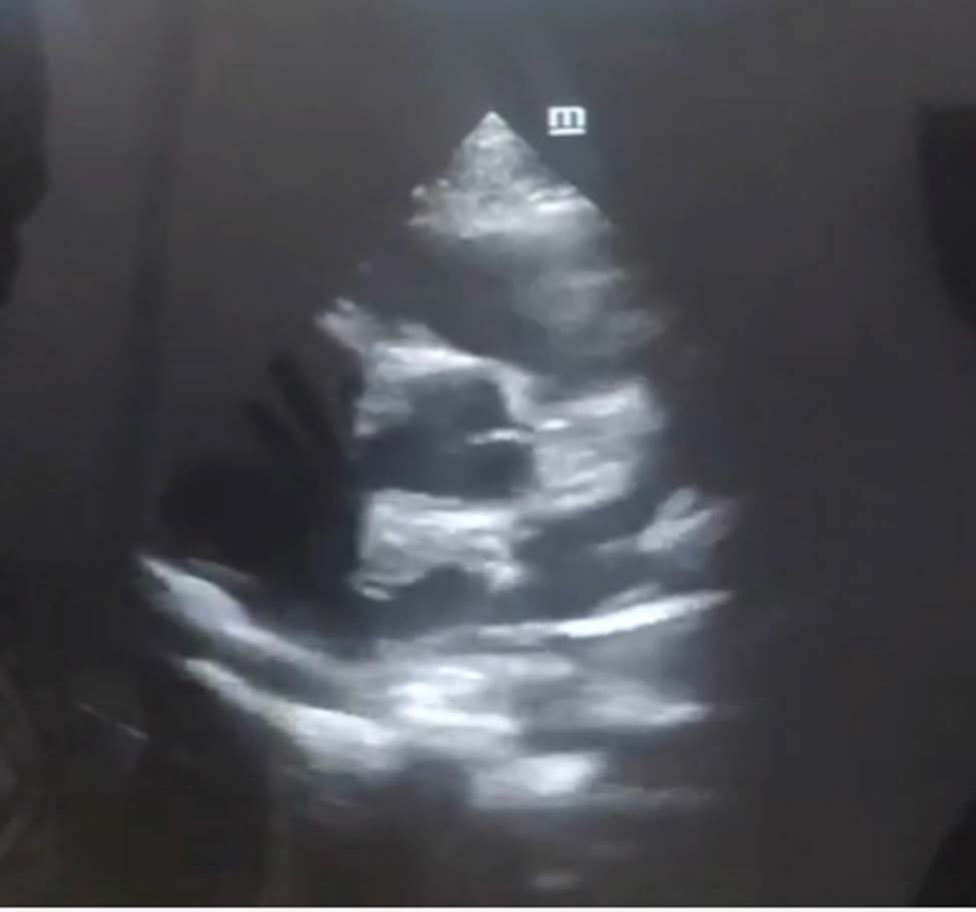
Echocardiographic image showing large embolus in the main pulmonary artery.

**Figure 4.**
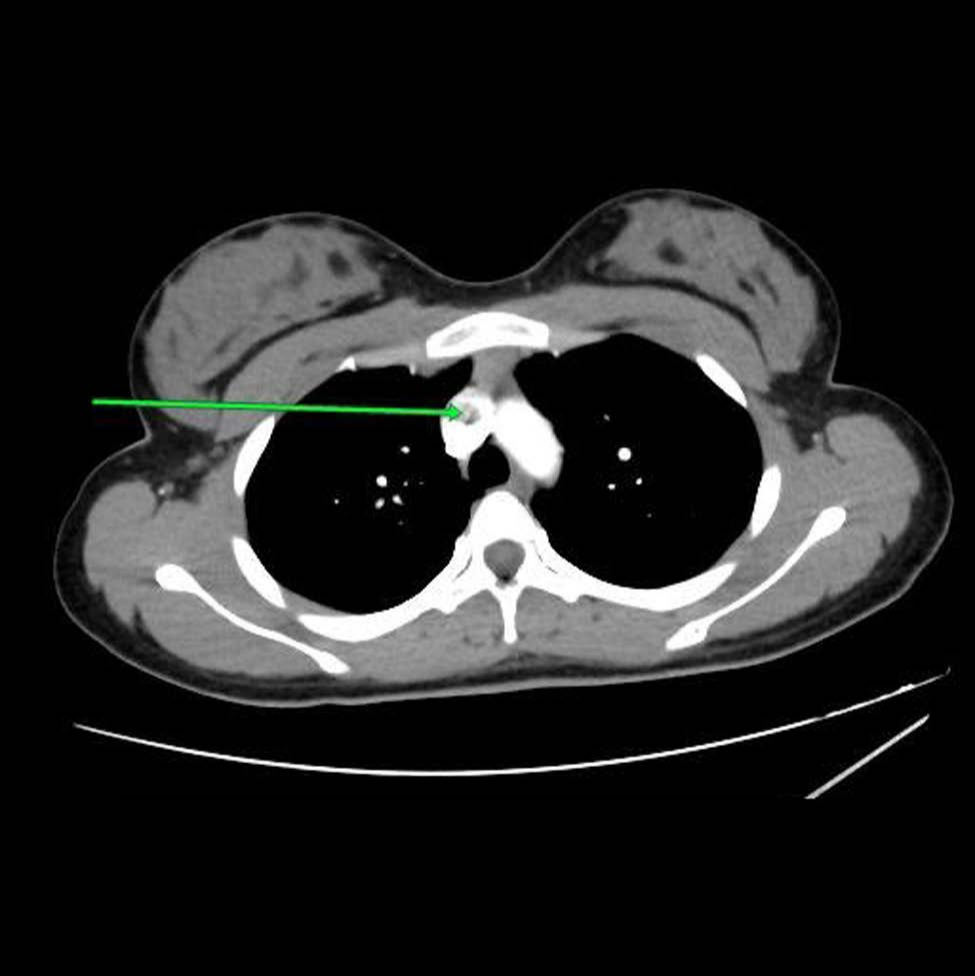
Axial section of a computed tomography pulmonary angiogram at the level of the main pulmonary vessels showing partial filling defects in the main right pulmonary artery as highlighted with the arrow.

**Figure 5.**
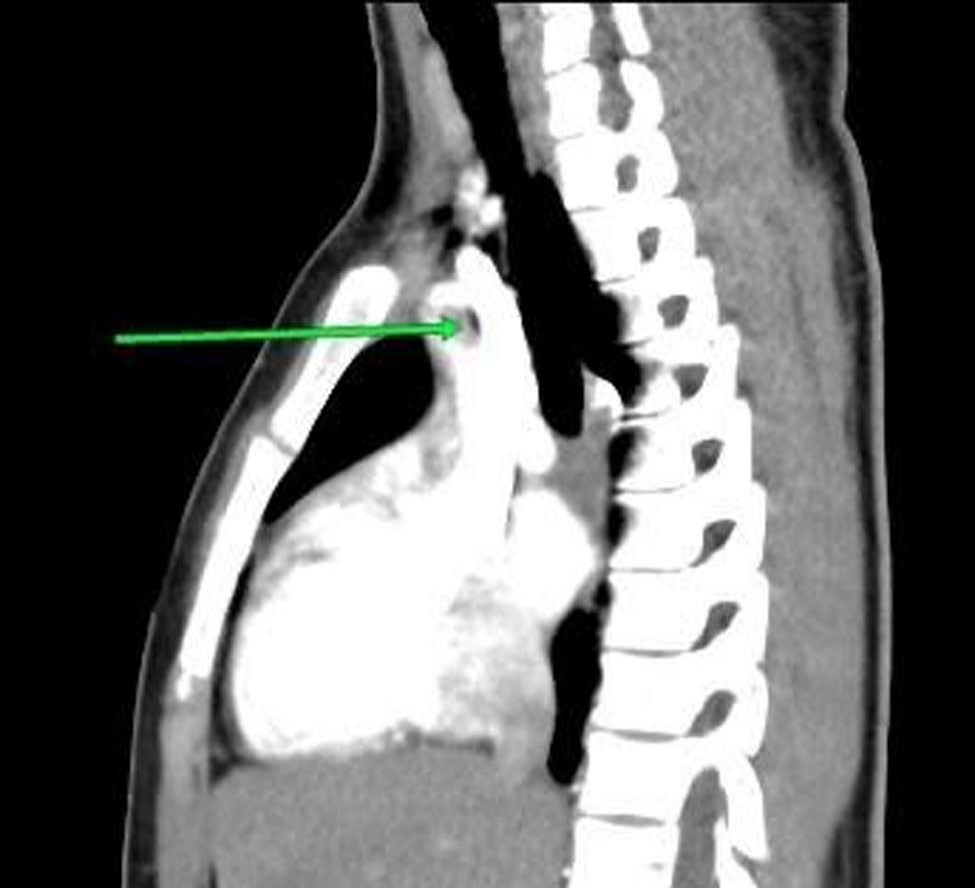
Sagittal section of a computed tomography at the level of the main pulmonary vessels showing a filling defect in the main right pulmonary as highlighted with the arrow.

She was admitted for suspected pulmonary embolism and managed by a multi-disciplinary team of pediatric and adult cardiologists, intensivists, radiologists and hematologists. The patient was jointly managed by specialists drawn from public tertiary hospitals. Due to the unavailability of some diagnostic services at the private hospital, the patient was subsequently referred to the State-owned tertiary hospital for hematological, chemical pathology, and radiological investigations, returning afterwards for continued care. Investigations done showed elevated D-dimer (>0.5 mg/dL). Platelet count, clotting profile, and liver function tests were normal. The chest radiograph was normal. An electrocardiogram showed evidence of bi-ventricular hypertrophy and sinus arrhythmia (Fig. 1). Echocardiography revealed a 4 mm ostium secundum atrial septal defect (ASD), and a huge thrombus at the main pulmonary trunk (Figs. 2 and 3), but the ventricular function was normal. Chest computed tomography appeared normal. On computerized tomography pulmonary angiogram (CTPA), partial filling defects in the main pulmonary artery and superior vena cava (Figs. 4 and 5) were seen in 2 consecutive images. Doppler ultrasound scan was normal. She was managed as a case of confirmed pulmonary embolism. Pharmacological therapy was commenced, and she was placed on low molecular weight heparin, sub-cutaneous Clexane at 40 μg daily for 5 days, and a stat dose of recombinant tissue plasminogen activator. Additionally, Alteplase infusion was given at 0.75 μg/kg IV over 2 hours.

A repeat echocardiogram done a week after anticoagulation therapy showed a reduction in the size of the thrombus. She was discharged home on a direct factor Xa inhibitor: oral Rivaroxaban 15 mg twice daily for 3 weeks, and then 20 mg daily for 3 months. The serial echocardiography showed gradual resolution of the embolus over 3 months of follow-up. Patient was adherent to medications, as parents directly observed and recorded intake.

Some diagnostic limitations were encountered; investigations like the chest computed tomography, CTPA and D-dimer were done in a public referral center, making the coordination of care cumbersome. In addition, being the only State Government-owned tertiary facility with such services and an enormous patient load, patient made multiple visits, with delays in scheduling appointments and retrieving results. The financial burden of care was huge and could have been a considerable limitation for most of the Nigerian population. However, the patient was on a private health insurance with extensive coverage, further buttressing the importance of healthcare financing arrangements.

### Patient consent:

The head of the managing team takes full responsibility that the patient details have been anonymized. Parents of the patient also gave an informed consent for the inclusion of their clinical and imaging details for this publication.

### Limitations and strengths:

Access to diagnostic services was a challenge; however, the managing team was able to coordinate multi-center and multi-disciplinary care. We were unable to carry out investigations for congenital coagulopathies because there were no facilities providing such service in the State. Alternatively, clotting studies were done with findings within normal limits. Unidentified confounding variables could have impacted our patient’s condition and management. Reporting bias is also a typical limitation of case reports, to avoid this we have used the standardized format and CARE guidelines to ensure consistency and thoroughness. In addition, we also reported negative findings where applicable.

## 3. Discussion

Pulmonary embolism is a potentially life-threatening condition, which is relatively uncommon in children and often presents with nonspecific symptoms.^[2,3]^ Most of the symptoms mimic common childhood illnesses and it may go unrecognized.^[3]^ The incidence of pulmonary embolism is reported to be 0.14–0.9/100,000, with a 2 to 3 times higher incidence in black children.^[3,4]^ These numbers may be underestimated due to the silent nature of pulmonary embolism in children and the common discovery of pulmonary emboli on autopsy studies of unsuspected cases.^[5]^ Interestingly, our case was the first reported in the center, this could possibly be attributed its rarity in children and adolescents, and partly to the dearth of advanced diagnostic services. It has a bimodal peak in the neonatal period and adolescence.^[2,6,7]^ Neonates have a higher incidence due to placement of central venous lines.^[8]^

Pulmonary embolism occurs following infections, congenital heart diseases, cancers, inherited and acquired thrombophilia and central line placements.^[9,10]^ Other risk factors include sedentary life style, recent surgery, and major trauma.^[11]^ Pulmonary embolism has been previously reported in a child with SARS COV 2, and a woman with atrial septal defect (ASD) where there was a left atrial thrombus that dislodged and caused pulmonary embolism by passing through the ASD.^[12–14]^ Regarding the possible causality, our patient incidentally had an undiagnosed congenital heart defect; a 4 mm ostium secundum ASD was noted during echocardiography. Although, the exact mechanisms for thrombi formation are unknown, an increased risk is reported, especially in the setting of pulmonary hypertension. This patient had no history suggestive of sepsis, malignant disease, or known congenital coagulopathies. A screening for coagulopathies was indicated in this patient, however, it was impossible to carry out, as no such facilities were available.

Tachycardia, tachypnea, pedal edema, and signs of right-sided heart failure are the classical signs of pulmonary embolism.^[3]^ Other symptoms include hemoptysis, shortness of breath, pleuritic chest pain, cough, and syncope.^[15]^ A high index of suspicion was helpful in this case, as our patient presented with some typical symptoms including tachycardia, palpitations, chest pain, and shortness of breath. Although, there was no peripheral edema and heaving apex, echocardiography showed biventricular hypertrophy. Pulmonary embolism usually goes undiagnosed due to its nonspecific symptoms and it takes approximately 7 days to make an accurate diagnosis.^[3]^ An electrocardiogram may show evidence of right atrial dilatation, right bundle branch block, sinus tachycardia and ST segment anomalies.^[6]^ The index patient had evidence of ST segment anomalies on electrocardiogram, and we posit that this could have resulted from right ventricular (RV) ischemia. Such ischemia could possibly be due to an increase in right ventricular afterload, thereby causing the dilatation and biventricular hypertrophy seen during echocardiography. Our patient’s chest pain can be explained by a possible RV ischemia triggering a release of vasoactive cytokines, and increased interstitial fluid in the lungs.

Although Biss et al^[16]^ showed a low utility of D-dimer in children, it has been proven that D-dimer can safely exclude pulmonary embolism in patients with a low clinical probability.^[3]^ D-dimer was elevated (>0.5 mg/dL) in our patient. CTPA is a quick and reliable way to delineate the presence and extent of pulmonary embolism, but there is a risk of exposure to ionizing radiation and it is insensitive to small emboli.^[3]^ The CTPA done for this patient showed filling defects in the main pulmonary artery and superior vena cava. Magnetic resonance angiography is a safer option as it eliminates the risk of ionizing radiation, but its use has not been fully studied in children.^[3]^

Thrombolysis may be done pharmacologically or mechanically.^[17]^ Recombinant tissue plasminogen activator is the recommended treatment for medical thrombolysis, and it can be given through a peripheral vein. A central catheter-directed method or surgical thrombolysis may be used for patients at risk for bleeding. Anti-coagulation may be continued for 3 to 12 months, and the patient should be followed up for years to determine resolution, progression or recurrence.^[3]^ In this case we deployed pharmacological therapy successfully. Our management therefore, provides evidence of the effective and safe use of pharmacotherapeutics in managing pulmonary embolism.

This case is unique for a number of reasons, particularly in our setting Nigeria. Nigeria suffers chronic workforce shortages exacerbated by physician emigration.^[18]^ Health services are inequitably distributed and specialist are concentrated in government tertiary hospitals available in urban areas. Majority of the population are without health insurance coverage and patronize private health facilities.^[18]^ Such centers may offer more conducive and personalized care, but are often more expensive and are limited in the range of services they can provide. The patient in our case suffered from a rare condition that could have been easily misdiagnosed or difficult to confirm either due to unavailability of laboratories and radio-imaging centers with capabilities tailored to pulmonary embolic events. This can be contributory to the reduced prevalence in settings like Nigeria, and other low-and middle-income countries. There were potential instances for delay in seeking-care which could have been deleterious for a life-threatening condition requiring emergency interventions to abort further complications. The logistics of traveling to satellite centers for diagnostic evaluations and moving back to the primary managing facility can be cumbersome for some families. In addition, without reasonable health insurance coverage, optimal care could have been impeded.

*Patients’ (parents) perspective:* We were concerned about the sudden chest pain, although we initially thought it was going to improve, but, it became worse, and we rushed her to the hospital when she started crying. We are grateful to the medical team for the prompt escalation of her case immediately they examined and suspected it was a pulmonary embolism. Several team members counseled us and provided a list of options to enable them to confirm their diagnosis. Most investigations were expensive, and we consider ourselves lucky to enjoy health insurance coverage. We were also fortunate that the radiology suite was recently commissioned by the state government and had all the options we used. Previously, one would have arranged for transportation to distant private diagnostic facilities where the services are more expensive. Overall, we were satisfied with the care, and our daughter is healthy and active.

## 4. Conclusion

Pulmonary embolism is a rare condition in children, and it is essential to have a high degree of suspicion to arrive at the correct diagnosis. It is also important to improve the diagnostic evaluation and multi-disciplinary intervention capacity. This can be challenging in low-resource settings where specialized modalities may be unavailable or unaffordable to clients. Early diagnosis and timely intervention are crucial in saving lives. This case also adds to the growing evidence of the effective role of pharmacological therapy in the management of pulmonary embolism.

## Author contributions

**Conceptualization:** Obuoma Umejuru Amaewhule.

**Data curation:** Obuoma Umejuru Amaewhule, Faithful Miebaka Daniel.

**Investigation:** Obuoma Umejuru Amaewhule, Ebbi Donald Robinson, Ugoeze Nneka Iloeje, Emmanuel Ovundah Nyeche.

**Project administration:** Obuoma Umejuru Amaewhule, Victoria Ezinne Emeruwa, Faithful Miebaka Daniel.

**Resources:** Ugoeze Nneka Iloeje, Emmanuel Ovundah Nyeche, Faithful Miebaka Daniel.

**Supervision:** Obuoma Umejuru Amaewhule.

**Validation:** Obuoma Umejuru Amaewhule, Ebbi Donald Robinson, Ugoeze Nneka Iloeje, Emmanuel Ovundah Nyeche, Victoria Ezinne Emeruwa, Faithful Miebaka Daniel.

**Writing – original draft:** Obuoma Umejuru Amaewhule, Faithful Miebaka Daniel.

**Writing – review & editing:** Faithful Miebaka Daniel, Victoria Ezinne Emeruwa.
